# Human papillomavirus vaccine acceptance among adolescent girls in Ethiopia: a systematic review and meta-analysis

**DOI:** 10.1186/s12889-023-16305-3

**Published:** 2023-07-17

**Authors:** Amare Zewdie, Abebaw Wasie Kasahun, Adane Habtie, Anteneh Gashaw, Mulat Ayele

**Affiliations:** 1 Department of Public Health, College of Medicine and Health Science, Wolkite University, Wolkite, Ethiopia; 2 Department of Midwifery, College of Medicine and Health Science, Dilla University, Dilla, Ethiopia; 3 Department of Midwifery, College of Medicine and Health Science, Woldia University, Woldia, Ethiopia

**Keywords:** Adolescent girls, HPV vaccination, Cervical cancer, Systematic review and meta-analysis, Ethiopia

## Abstract

**Background:**

Cervical cancer is the fourth most common cancer affecting females. Human papillomavirus vaccination of adolescent girls is the primary strategy for cervical cancer prevention but in Ethiopia, it lacks emphasis. Despite different studies done and found a highly variable level of vaccine acceptance; however, there was no summarized evidence on the issues as a nation. Thus this systematic review and meta-analysis aimed to assess the pooled prevalence of human papillomavirus vaccine acceptance by adolescent girls and its associated factors in Ethiopia.

**Method:**

A systematic review and meta-analysis were conducted using PRISMA guidelines. Comprehensive literature was searched in PubMed, Google Scholar, and African Online Journal databases. A weighted inverse variance random effect model was used to estimate pooled prevalence. Cochrane Q-test and I^2^ statistics were computed to assess heterogeneity among studies. Funnel plot and Eggers test were done to assess publication bias. Review manager software was used to identify factors associated with vaccine acceptance.

**Result:**

Overall, 157 articles were retrieved and finally 7 articles were included in this review. The pooled prevalence of adolescent human papillomavirus vaccine acceptance was 46.52% (95%CI; 30.47-62.57%). Subgroup analysis showed that adolescent vaccine acceptance was highest in the Oromia region and lowest in Addis Ababa. Knowledge about human papillomavirus vaccination (AOR = 3.89, 95% CI: (2.85–5.32)) and attitude (AOR = 2.65, 95% CI: (2.03– 3.44)) were significantly associated with adolescent’s vaccine acceptance.

**Conclusion:**

Human papillomavirus vaccine acceptance of adolescent girls in Ethiopia was low. Knowledge about the vaccine and attitude to vaccination were positively associated with their vaccine acceptance. Therefore, policymakers and program planners should target school-aged adolescents in increasing their awareness and changing their attitudes to enhance their vaccine acceptance in order to prevent and control cervical cancer.

**Supplementary Information:**

The online version contains supplementary material available at 10.1186/s12889-023-16305-3.

## Background

Following breast, colorectal, and lung cancers, cervical cancer is the fourth most common cancer affecting females [1]. Globally, in 2020, there were an estimated 604,127 cases and about 341,831 deaths of cervical cancer in which 85% of whom were from the world’s poorest countries [2]. The highest regional morbidity and mortality of this cancer is in Sub-Saharan Africa where it stands second behind breast cancer [3]. Ethiopia as part of the sub-Saharan region shares the regional high incidence and mortality of cervical cancer which makes it one of the major public health problems. It ranks second in cancer-related mortality next to breast cancer among all women and third cause of cancer deaths among reproductive-age women [4]. Despite, it is a preventable form of cancer both the incidence and mortality figures in national as well as global contexts reflect it as a major reproductive health problem that affects the quality of life of women.

An estimated 90% of cervical cancer cases were associated with human papillomavirus (HPV) infection [5]. HPV is a sexually transmissible, non-enveloped, double-stranded DNA virus and identified as the commonest cause of oral, oropharyngeal, vulvar, vaginal, and cervical cancers and genital warts [6]. HPV serotype16, 18, and 31 were the most frequently detected serotypes as a cause of cervical cancer [7]. Infection with those cancerous serotypes results in precancerous cervical lesions which later progress to advanced malignancy; while infection with other serotypes can be cleared by the host immune system [8]. Around 80% of sexually active women are at risk of contracting Human Papilloma Virus [9].

Cervical cancer is preventable cancer through highly effective HPV vaccine which prevents the commonest strains of the virus. The first safe and efficacious HPV vaccine was developed in 2006 [10]. HPV vaccination can prevent more than 90% of cervical cancer cases [11]. As of 2020, more than half of the WHO member countries have introduced HPV vaccination programs to meet the 2030 sustainable development goal elimination target of 90% [12]. Evidence shows that the introduction of this HPV vaccine resulted in a reduction in persistent HPV infection and cervical lesions in several countries. HPV vaccination offers an opportunity for developing nations to decrease the burden of cervical cancer [13].

However Globally, only 39.7%, of women are vaccinated against HPV while this figure is 68% in high-income countries and 2.7% in lower-middle-income countries [14]. Ethiopia introduced the HPV vaccine for girls who are 14 years of age in 2018. Then the vaccine is delivered in a School-based approach for those target groups however Ethiopia as a member of low-income countries shares lower coverage of HPV vaccination. For out-of-school girls, the vaccine is planned to be given at health facilities in all 11 regions and the two city administrations of the country [15].

Furthermore, Vaccination of vulnerable girls and women in the country faces several challenges. Vaccine shortages and the problem of availability at all health facilities, long-rooted cultural beliefs on the cause and treatment of cervical cancer such as considering the cause of the disease as a breaching of social taboos, and a lack of community engagement to raise awareness about cervical cancer and lack of commitment in the health sector to consider it as routine task were the most common identified challenges in the country in the implementation of HPV mass vaccination [16, 17]. Such challenges in the execution of this primary prevention strategy of cervical cancer make sub-Saharan Africa which includes Ethiopia, the greatest cervical cancer mortality region of the world.

So far, in Ethiopia, different studies have been done on HPV vaccine acceptance of adolescent girls and found a highly variable level of acceptance across the regions of the country. For example vaccine acceptance in South nation nationality and peoples region (SNNPR) was 50.39% [18], ranges 16.45–66.5% in Amhara [19, 20], 44.2–68.85% in Oromia [21–23] and 30.03 in Addis Ababa [24]. However, up to the level of our knowledge, there is no systematic review and meta-analysis done in Ethiopia which can provide summarized evidence on HPV vaccine acceptance of adolescents; despite this being the main area of intervention focus which increase the need to summarize the issue and intervene accordingly. Because annually, an estimated 6,300 new cervical cancer cases are diagnosed and 4884 deaths occur in the country which calls action to improve preventive strategies such as HPV vaccination of adolescent girls [25]. Thus, this systematic review and meta-analysis aimed to assess the pooled prevalence of HPV vaccine acceptance and identify its associated factors among Ethiopian adolescent girls.

### Significance of the study

The evidence from this systematic review and meta-analysis can be used for planning and implementation of an intervention to improve HPV vaccination in adolescent girls. It identifies the important factors that affect vaccine acceptance, therefore it contributes evidence input for preparing messages and materials for outreach and media campaigns to increase vaccine uptake and so as to prevent cervical cancer. This study can also serve as a baseline comparison since there is no systematic review and meta-analysis done on this topic. In addition, it may ignite new insight for further studies that might be conducted on related topics.

## Method

### Study design and setting

A systematic review and meta-analysis were conducted on HPV vaccine acceptance among adolescent girls in Ethiopia. Preferred Reporting Items for Systematic Review and Meta-Analysis guidelines were followed (Supplementary Table 1). PRISMA is a protocol consisting of checklists that guide the conduct and reporting of systematic reviews and meta-analyses, which increase the transparency and accuracy of reviews in medicine and other fields [26]. Ethiopia is one of the low-income countries located in the Horn of Africa with a 2022 projected population of 123.4 million, 133.5 million in 2032, and 171.8 million in 2050 [27]. For administrative purposes, Ethiopia is divided into 11 regions and 2 city administrations. Regions are further classified into zones, and zones are divided into districts. Finally, districts are divided into kebele (the smallest administrative division contains 2000 up to 3500 residents).

### Search strategies and sources of information

We have checked the PROSPERO database (http://www.library.ucsf.edu/) whether published or ongoing projects exist related to the topic to avoid any further duplication. Thus, the findings revealed that there were no ongoing or published articles in the area of this topic. Then this systematic review and meta-analysis were registered in the PROSPERO database with Id no CRD42023387660. Comprehensive literature was searched using international databases PubMed, Google Scholar, and African Online Journal to retrieve related articles. Grey literature was searched using Google. Search terms were formulated using PICO guidelines through online databases. Medical Subject Headings (MeSH) and key terms had been developed using different Boolean operators ‘AND’ and ‘OR’. The following search term was used: “Human papillomavirus vaccine” OR “HPV vaccine” OR “cervical cancer preventive vaccine” AND “acceptance” OR “uptake” OR “utilization” AND “adolescent girls” OR “school-aged adolescents” OR “school girls” OR “female students” AND Ethiopia.

### Eligibility criteria

To be included in this systematic review and meta-analysis, studies should be on HPV vaccine acceptance of adolescent girls and its determinants in the English language, without restriction on race, or publication date (until the last search date January 30, 2023). Since there were no other reviews on the topic and HPV vaccination is a new or current agenda which initiated in 2018 in Ethiopia, therefore we decide to include studies from any time period. Articles without full abstracts or texts and articles reported out of the outcome interest were excluded. Citations without abstracts and/or full-text, commentaries, anonymous reports, letters, editorials, reviews, and meta-analyses were excluded at each respective stage of screening.

### Outcome measurements

This study has two main outcomes. The primary outcome was the magnitude of HPV vaccine acceptance among adolescent girls. It is defined as the proportion of participants who accept or are willing to vaccinate against HPV. Therefore, all included studies asked the study participants about their acceptance and categorized them as willing to accept the vaccine or not. Then, the response was analyzed and presented as the prevalence of HPV vaccine acceptance among adolescent girls. The secondary outcome was determinants of HPV acceptance of adolescent girls.

### Data extraction

All studies obtained from the considered databases were exported to Endnote version X8 software to remove duplicate studies. Then after, all studies were exported to a Microsoft Excel spreadsheet. All authors independently extracted the important data using a standardized data extraction form which was adapted from the Joanna Briggs Institute (JBI) data extraction format. For the first outcome (prevalence) the data extraction format included (primary author, year of publication, regions, study area, sample size, and prevalence with 95% CI). We extracted data for the second outcome (associated factors to HPV vaccine acceptance) using a 2 by 2 table format. Finally, the log odds ratio for each factor was calculated using Review Manager (RevMan) software 5.4.

### Quality assessment

To assess the quality of each study included in this systematic review and meta-analysis, the modified Newcastle Ottawa Quality Assessment Scale for cross-sectional studies was used [28] (Supplementary Table 2). Two Authors (AZ, AWK) assessed the quality of each study (i.e. methodological quality, sample selection, sample size, comparability and the outcome, and statistical analysis of the study). In the case of disagreement between two authors; another three authors (AH, AG, MA) were involved and discussed and resolved the disagreement.

### Data processing and analysis

The extracted Microsoft Excel spreadsheet format data was imported to STATA version 14 for analysis. Then weighted inverse variance random effect model was used to estimate the pooled prevalence of HPV vaccine acceptance among adolescent girls in Ethiopia. Cochrane Q-test and I^2^ statistics were computed to assess heterogeneity among all studies. Accordingly, if the result of I^2^ is 0–40% it is mild heterogeneity, 40 to 70% would be moderate heterogeneity, and 70 to 100% would be considerable heterogeneity [29]. Funnel plot and Eggers test were done to assess publication bias. The p-value > 0.05 indicated that there was no publication bias. Subgroup analysis was done based on the study region. A forest plot format was used to present the pooled prevalence of HPV vaccine acceptance of adolescent girls with 95%CI. To identify determinants of HPV vaccine acceptance in adolescent girls, we have used review manager software.

### Result

**Fig. 1:**
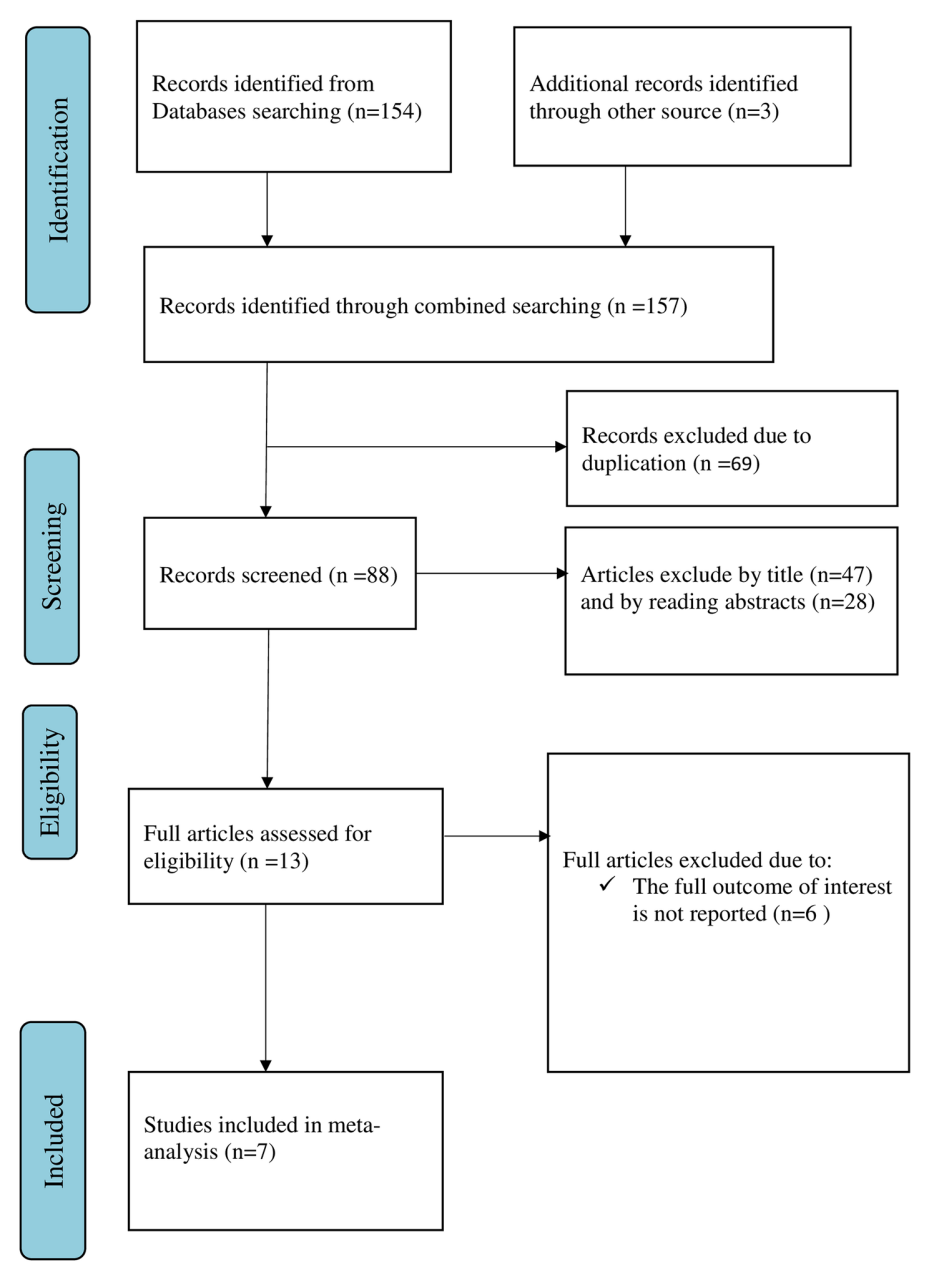
; Flow chart of selection for systematic review and meta-analysis on HPV vaccine acceptance among adolescent girls and its determinant in Ethiopia, 2023

A total of 157 articles were found using our search strategy in African Journals Online, Google Scholar, and PubMed databases. 88 articles were left after the removal of duplicates (69). Then 47 and 28 articles were excluded by reviewing the titles and the abstracts respectively. As a result, 13 full-text papers were accessed, evaluated for inclusion criteria, and 6 more articles were excluded for the aforementioned reason. Therefore, 7 papers were eligible for inclusion in the final systematic review and meta-analysis (Fig. 1).

Of the included studies in this SRMA, three were done in Oromia, two in Amhara, one in Addis Ababa, and one in the SNNPR. All included studies were cross-sectional. They included a total of 3315 participants which ranges from 366 to 633 participants per study and found 30–68.9% HPV vaccine acceptance among adolescent girls. Regarding the quality of included studies, the Newcastle Ottawa Quality Assessment scale score of all included studies lies from 8 to 9 which is good (Table 1).

**Table 1:** Characteristics of included studies in the systematic review and meta-analysis on HPV vaccine acceptance among adolescent girls in Ethiopia

s.no	Author	Year	Region	study design	Sample	Prevalence of acceptance %	quality
1	Ukumo, et al.(18)	2020	SNNPR	Cross-sectional	516	50.39	Good
2	Lakneh, et al.(19)	2022	Amhara	Cross-sectional	620	16.45	Good
3	Kassa, et al.(20)	2020	Amhara	Cross-sectional	591	66.5	Good
4	Beyen, et al.(21)	2020	Oromia	Cross-sectional	414	44.2	Good
5	Biyazin, et al. (22)	2022	Oromia	Cross-sectional	366	68.85	Good
6	Geneti, et al.(23)	2019	Oromia	Cross-sectional	397	49.37	Good
7	Regasa T.(24)	2022	Addis Ababa	Cross-sectional	393	30.03	Good

### Magnitude HPV vaccine acceptance among adolescent girls in Ethiopia

**Fig. 2:**
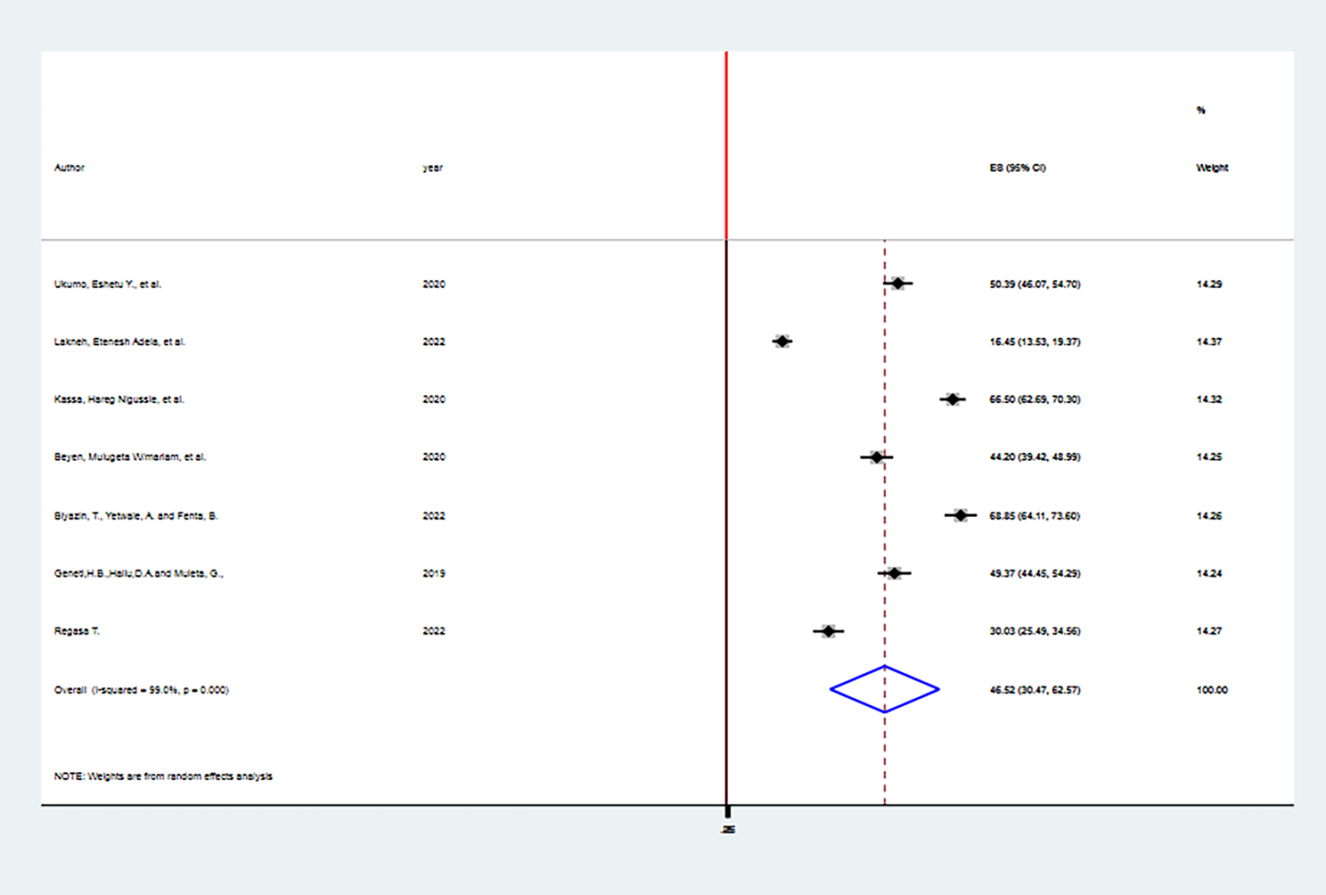
; The Pooled prevalence of HPV vaccine acceptance among adolescent girls in Ethiopia, 2023

The pooled prevalence of HPV vaccine acceptance among adolescent girls in Ethiopia was 46.52% (95%CI; 30.47-62.57%), with the Cochrane heterogeneity index (I^2^ = 99.0%), P = 0.000, showing substantial heterogeneity of different studies (I^2^ > 70%). Therefore we have used the random effect model to resolve the issue of heterogeneity among included studies. Additionally, we have considered subgroup analysis as a potential way of addressing heterogeneity. The finding was presented using a forest plot (Fig. 2).

### Publication bias

**Fig. 3:**
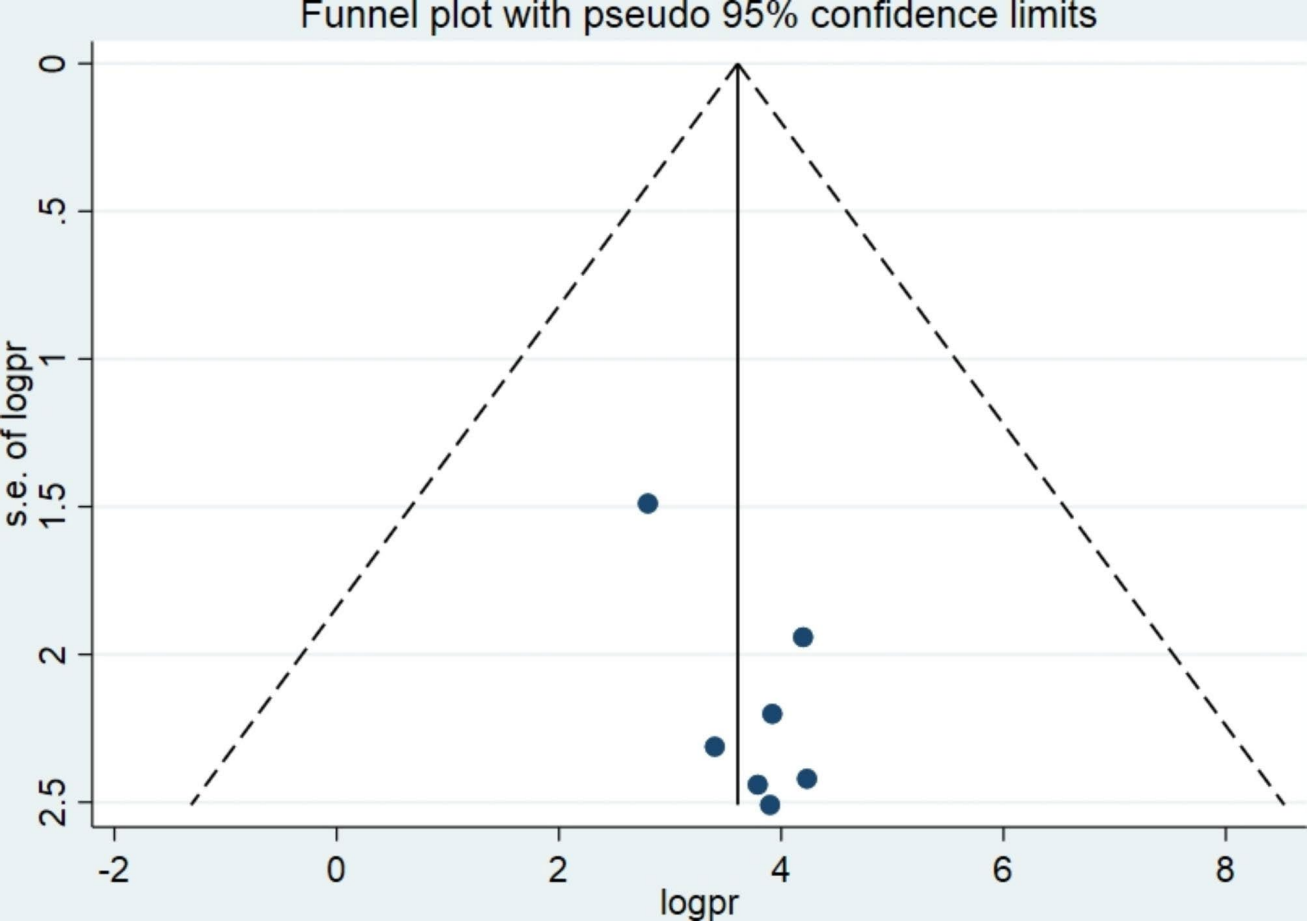
; Funnel plot showing the symmetric distribution of articles on HPV vaccine acceptance among adolescent girls in Ethiopia, 2023

In this systematic review and meta-analysis, the statistical analysis (funnel plot and Egger’s regression test P = 0.056 (p > 0.05)) result showed no publication bias (Fig. 3). However the result of the test might be affected by the small number of included studies and the substantial heterogeneity between included studies.

### Subgroup analysis of HPV vaccine acceptance among adolescent girls in Ethiopia

**Fig. 4:**
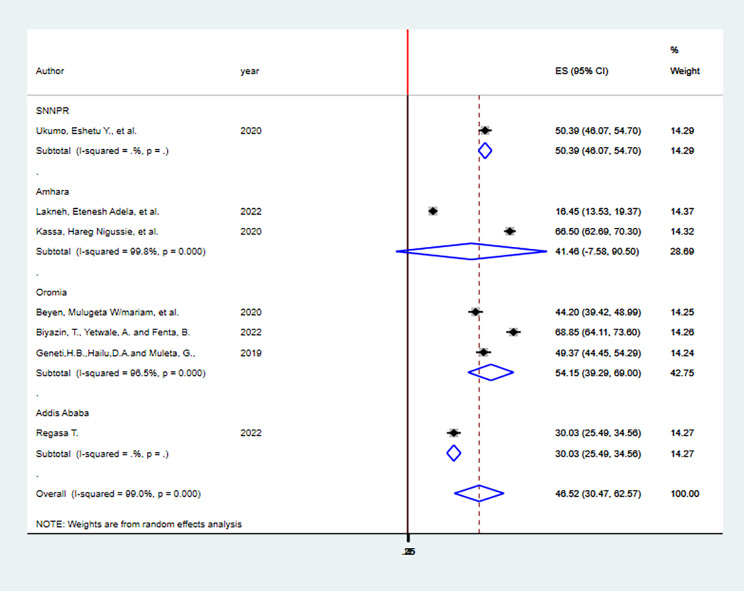
; Forest plot showing Subgroup analysis of HPV vaccine acceptance among adolescent girls in Ethiopia, 2023

The finding of subgroup analysis by region showed that the pooled prevalence of HPV vaccine acceptance among adolescent girls was highest in Oromia region (54.15%; 95% CI: (39.29-69.00), I^2^ = 96.5%, p = 0.000) followed by SNNPR (50.39%; 95% CI: (46.07–54.70), I^2^ = 0%, p = 1) then Amhara region (41.46%; 95% CI: (7.58–90.50), I^2^ = 99.8%, p = 0.000) and least Addis Ababa(30.03%; 95% CI: (25.49–34.56), I^2^ = 0%, p = 1) (Fig. 4).

### Sensitivity analysis

**Fig. 5:**
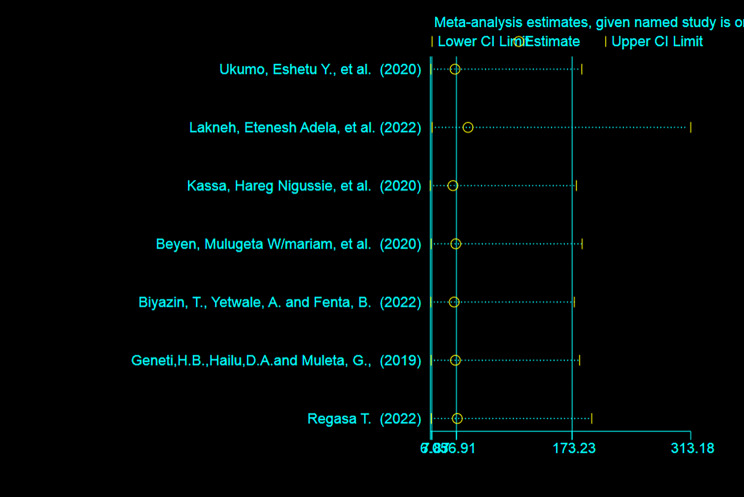
; Sensitivity analysis of HPV vaccine acceptance among adolescent girls in Ethiopia, 2023

A random-effect model result showed that; no single study has influenced the overall pooled prevalence of HPV vaccine acceptance among adolescent girls in Ethiopia (Fig. 5).

### Determinants of HPV vaccine acceptance among adolescent girls in Ethiopia

In our systematic review and meta-analysis; two factors that are associated with HPV vaccine acceptance in two or more primary studies are included in the identification of the determinants of HPV vaccine acceptance among adolescent girls. Accordingly, knowledge about HPV vaccination and attitude were significantly associated with HPV vaccine acceptance among adolescent girls in Ethiopia. Adolescent girls who have good knowledge about the HPV vaccine were 3.89 times more likely to accept the HPV vaccine as compared to their counterparts (AOR = 3.89, 95% CI: (2.85–5.32)). Similarly, Adolescent girls who have a positive attitude to the vaccine were 2.65 times more likely to accept the HPV vaccine as compared to girls who have a negative attitude (AOR = 2.65, 95% CI: (2.03– 3.44)) (Table 2).

**Table 2:** Factors associated with HPV vaccine acceptance among adolescent girls in Ethiopia

Variable	Authors	AOR	95%CI	Pooled AOR	95%CI of pooled AOR
Good knowledge	Ukumo, et al.	2.49	1.19_ 5.24	3.89	2.85–5.32
	Lakneh, et al.	2.36	1.48_3.76		
	Kassa, et al.	8.65	5.2_14.3		
Positive attitude	Beyen, et al.	2.05	1.15_3.64	2.65	2.03– 3.44
	Ukumo, et al.	5.22	2.96_9.19		
	Lakneh, et al.	2.87	1.7_4.85		
	Kassa, et al.	1.85	1.18_3		

Where; AOR: is the odds ratio of the respective variable in each primary study

Pooled AOR: is the point value of odds ratio when we pooled the AOR of primary studies by our analysis

95%CI of pooled AOR: is the 95%CI of the point value of pooled AOR that is the output of our analysis

## Discussion

HPV vaccination is a proven and effective strategy in the prevention of cervical cancer and its consequences. Despite the HPV mass vaccination of adolescent girls being the primary prevention of precancerous cervical lesions of HPV infection which was approved and initiated by WHO, however, its implementation is much lower, especially in developing countries [30]. In Ethiopia, HPV vaccination has been given in school-based campaigns and which needs great inter-sectoral collaboration and coordinated effort to improve its coverage so as to reduce the burden of cervical cancer in the country. The current systematic review and meta-analysis aimed to assess the magnitude and determinants of HPV vaccine acceptance among adolescent girls in Ethiopia.

This review found a 46.52% (95%CI; 30.47-62.57%) pooled prevalence of HPV vaccine acceptance among adolescent girls in Ethiopia. The finding was consistent with a study finding from Uganda 42.4% [31]. However, this was extensively higher than the study conducted in Ibadan, Nigeria (4.1%) [32]. The possible justification for the discrepancy may be the Nigeria study was conducted among a small sample size and also it was conducted before 2017 in which HPV vaccination is not much promoted which makes the prevalence of vaccine acceptance low. Our review finding is much lower than from studies done in developed countries, such as Scotland (94.4%), Taiwan (91%), Canada (87.4%), Australia (83%), Denmark (80%), and Malaysia (77.9%) [33]. The difference from those studies might be due to the vaccine accessibility; as these countries have better access coverage. They had also implemented routine HPV vaccination for all eligible girls. This visible discrepancy in the level of vaccine acceptance between developed and developing countries (our finding) reflects and answered the question of why cervical cancer becomes a great challenge for the world’s poorest countries. The finding implies that a strong effort should be made to increase HPV vaccine acceptance in adolescent girls so as to prevent recurrent HPV infection and its consequence which is cervical cancer.

The current review also identified the factor significantly associated with HPV vaccine acceptance in adolescent girls. Thus, adolescent girls who have good knowledge about the HPV vaccine were around four times more likely to accept the HPV vaccine as compared to adolescents who have poor knowledge about the vaccine. The finding was similar to studies conducted in Melaka, Malaysia [34], and Korean Americans [35] in which better adolescent knowledge about the vaccine was linked with increased vaccine acceptance. A better degree of awareness of the study participants regarding the importance of HPV vaccination, the danger of cervical cancer, and its consequences may account for the association between good knowledge and acceptance of the HPV vaccination. This evidence implies vaccination enhancement intervention should focus on improving awareness which can refute myths and misconceptions about the vaccine and then increase vaccine uptake among adolescent girls. The finding also calls to action to the local health sector to strengthen their inter-sectoral collaboration with primary and secondary schools as well as with universities in awareness raising for adolescent girls about cervical cancer prevention strategies including HPV vaccination.

In the current review adolescent girls who have favorable attitudes are around three times more likely to accept the HPV vaccine as compared to their counterparts. The finding was in line with studies done in the Liara and Ibanda districts of Uganda[36, 37], Canada [38], and China [39] in which favorable attitude was positively associated with adolescents’ HPV vaccine acceptance. This may be explained by the fact that most motivating factors of adolescents’ HPV vaccine utilization are generated from their positive attitude which can be concluded as the impact of attitude on behavior. This attitudinal belief is mainly driven by their feeling and beliefs regarding the vaccine’s effectiveness, safety, and compatibility with general societal beliefs. The finding implies great effort should be made in enhancing adolescent’s positive feelings and belief about the HPV vaccine by locally or domestically acceptable strategies regarding its safety and effectiveness which later improve their vaccine uptake. Furthermore, researchers are expected to explore in detail the various belief and long-rooted cultural values that might affect the attitude of adolescent girls as well as their parents using feasible qualitative approaches which can dig depth evidence about the issue.

Even though the necessary endeavors were made to minimize or avoid the possible limitations of this study, the result should be interpreted in light of some limitations. First, there were variations in outcome measurement (vaccine acceptance) among the included primary studies which might create confusion, and in our review, we tried to accommodate the outcome definition of all included studies. Secondly, even if the Egger’s test is not statistically significant there might be some sort of publication bias in our review because the power of Egger’s test is low when the number of included studies is small and if there is substantial heterogeneity. The absence of similar reviews in other countries makes it difficult to compare our findings with others and we are enforced to compare our findings with primary studies. Finally, our search strategy found limited studies (seven) especially no studies from Tigray, Dire Dawa, Afar, Gambella, Sidama, Hariri, Somali, and Benishangul-Gumuz regions.

## Conclusion

The pooled prevalence of HPV vaccine acceptance among adolescent girls in Ethiopia was low, which indicates that focused and carefully planned intervention should be designed and implemented to raise the level of willingness to accept and utilization of the vaccine so as to prevent and control the lethal cervical cancer. Knowledge about HPV vaccination and attitude were significantly associated with HPV vaccine acceptance in adolescent girls. Therefore, policymakers and program planners should focus on improving adolescent girl’s awareness and attitude about the HPV vaccine through a coordinated school and health system-based approach which in turn enhances their vaccine acceptance.

## Electronic supplementary material

Below is the link to the electronic supplementary material.

Supplementary Material 1

Supplementary Material 2

## Data Availability

The result of this SRMA was extracted from the data gathered and analyzed based on the stated methods and materials. All the relevant data are within the paper.

## Abbreviations

HPV: Human Papilloma Virus
PRISMA: Preferred Reporting Items for Systematic Reviews and Meta-Analyses
SNNPR: South Nation Nationality and People Region
SRMA: Systematic Reviews and Meta-Analyses
WHO: World Health Organization
