# Supplementary material for: Human papillomavirus vaccine acceptance among adolescent girls in Ethiopia: a systematic review and meta-analysis
Source: BMC Public Health. 2023 Jul 17;23:1369. doi: 10.1186/s12889-023-16305-3 (PMC10353090; doi:10.1186/s12889-023-16305-3)
Supplement: Supplementary file 2 — Supplementary Material 2 [file 12889_2023_16305_MOESM2_ESM.docx]

| **Supplementary table 2 :** Newcastle-Ottawa Quality Assessment Scale for cross sectional studies used in the systematic review and meta-analysis 2022 | | | | | | | | |
| --- | --- | --- | --- | --- | --- | --- | --- | --- |
|  | Selection | | | | Comparability | Outcome | | Total score |
| Authors | Representativeness s (1) | Sample size (1) | Non respondents (1) | Ascertainment of the exposure (risk factor) (2) | The subjects in different outcome groups are comparable, based on the study design or analysis. confounding factors are controlled (1) | Assessment of the outcome (2) | Statistical test (1) |  |
| Ukumo, Eshetu Y., et al.([21](#_ENREF_21)) | 1 | 1 | 1 | 2 | 1 | 2 | 1 | 9 |
| Lakneh, Etenesh Adela, et al.([22](#_ENREF_22)) | 1 | 1 | 1 | 2 | 1 | 2 | 1 | 9 |
| Kassa, Hareg Nigussie, et al.([23](#_ENREF_23)) | 1 | 1 | 1 | 2 | 1 | 2 | 1 | 9 |
| Beyen, Mulugeta W/mariam, et al.([24](#_ENREF_24)) | 1 | 1 | 1 | 2 | 1 | 1 | 1 | 8 |
| Biyazin, T., Yetwale, A. and Fenta, B.([25](#_ENREF_25)) | 1 | 1 | 1 | 2 | 1 | 1 | 1 | 8 |
| Geneti,H.B.,Hailu,D.A.and Muleta, G.,([26](#_ENREF_26)) | 1 | 1 | 1 | 1 | 1 | 2 | 1 | 8 |
| Regasa T.([27](#_ENREF_27)) | 1 | 1 | 1 | 2 | 1 | 1 | 1 | 8 |

The scoring process was made according to Newcastle-Ottawa Quality Assessment Scale adapted for cross sectional studies

**Selection: (Maximum 5 scores)**

**1) Representativeness of the cases:**

a) Truly representative of the HCC patients (consecutive or random sampling of cases). 1 score

b) Somewhat representative of the average in the HCC patients (non-random sampling) . 1 score

c) Selected demographic group of users. 0 score

d) No description of the sampling strategy. 0 score

**2) Sample size:**

a) Justified and satisfactory (≥ 400 HCC included). 1 score

b) Not justified (<400 HCC patients included). 0 score

**3) Non-Response rate**

a) The response rate is satisfactory (≥95%). 1 Score

b) The response rate is unsatisfactory (<95%), or no description. 0 Score

**4) Ascertainment of the screening/surveillance tool:**

a) Validated screening/surveillance tool. 2 scores

b) Non-validated screening/surveillance tool, but the tool is available or described. 1 score

c) No description of the measurement tool. 0 score

**Comparability: (Maximum 1 scores)**

1) **The potential confounders were investigated by subgroup analysis or multivariable analysis.**

a) The study investigates potential confounders. 1 score

b) The study does not investigate potential confounders. 0 score

**Outcome: (Maximum 3** scores**)**

**1) Assessment of the outcome:**

a) Independent blind assessment. 2 scores

b) Record linkage. 2 scores

c) Self report. 1 score

d) No description. 0 score

**2) Statistical test:**

a) The statistical test used to analyze the data is clearly described and appropriate. 1 score

b) The statistical test is not appropriate, not described or incomplete. 0 score
